# Association between obesity and atopic disorders in Chinese adults: an individually matched case–control study

**DOI:** 10.1186/1471-2458-13-12

**Published:** 2013-01-08

**Authors:** Xiao Luo, Jing Xiang, Xiaohui Dong, Fuwen Cai, Jianing Suo, Zhiqiang Wang, Meina Liu

**Affiliations:** 1 Public Health College, Harbin Medical University, 157 Baojian Road, Haerbin City, Heilongjiang Province, Postcode 150081, People's Republic of China; 2 School of Medicine, the University of Queensland, Room 817, Health Sciences Building, Royal Brisbane & Women’s Hospital, Herston, QLD 4029, Australia

## Abstract

**Background:**

Obesity is regarded as a potential risk factor for atopy. The aim of this study was to assess the associations of obesity with atopic dermatitis, rhinitis, asthma and food allergy in Chinese adults.

**Methods:**

Two hundred and sixty six (266) atopic cases in Harbin, China, were identified according to the current Chinese guidelines for the diagnosis of atopic diseases. All cases had a previous diagnosis of atopic disorders (atopic dermatitis, rhinitis, asthma or food allergy) and were positive in one or more allergen specific IgE tests to 16 common allergens in the region. Each case was individually matched to two healthy controls based on their age, sex, and residential regions. All 532 healthy controls were negative in allergen specific IgE tests. The associations of obesity with four atopic disorders were assessed using a conditional logistic regression method.

**Results:**

Obesity was significantly associated with the presence of atopic diseases (OR = 3.2, 95% CI: 1.8, 5.7). Males and females had a similar association (OR = 3.1 for males and 3.2 for females). The associations of obesity with atopic dermatitis (OR = 2.7, 95% CI: 1.2, 6.3) and atopic rhinitis (OR = 3.1, 95% CI: 1.1, 8.7) were statistically significant. Although obesity was positively associated with atopic asthma, this association was not statistically significant (OR = 3.4, 95% CI: 0.6, 19.9). The association between obesity and food allergy was weak and not significant (OR = 1.1, 95% CI: 0.4, 3.7).

**Conclusions:**

Obesity is positively associated with the presence of atopic diseases in Chinese adults. Specifically, obesity is significantly associated with atopic dermatitis and rhinitis. Our findings warrant further investigation on the causal nature between obesity and atopic diseases and the effect of weight reduction on preventing atopic diseases.

## Background

The prevalence of obesity has increased rapidly worldwide, particularly in low and middle income countries like China [1]. A number of diseases are related to obesity [2,3]. There is a current debate on the association between obesity and atopic diseases in adults. It is still not clear if the recent epidemic of obesity has contributed to the rise in the incidence of atopic diseases. Some studies showed a positive association [4-6] while others showed no association between obesity and atopic diseases in adults [7,8]. The incidence rates of atopic diseases, including atopic dermatitis, allergic rhinitis, allergic asthma, and food allergy, have increased in China recently [9]. Understanding the associations of obesity with those atopic diseases has important public health implications to elucidate the causal link and the importance of weight control in preventing atopic diseases.

Common allergens vary among different geographic regions and cultures [10], while the manifestations of atopy also vary among different populations [11-13]. Those variations may have contributed to the inconsistent findings in different study populations. The findings from other populations may not apply to Chinese adults. As obesity has become a major health concern in China, its associations with atopic diseases in this population are still not clear. In this individually matched case–control study of Chinese adults, we assessed the associations of obesity with four atopic diseases.

## Methods

### Study participants

This is a matched case–control study with a case to control ratio of 1:2. During March 2009 and March 2011, patients who visited the Department of Allergy of the First Affiliated Hospital of Harbin Medical University in Harbin in Northeast China were eligible for this study. Those with previously diagnosed atopic disorders were determined according to the current guidelines for allergic asthma [14], dermatitis [15], rhinitis [16] and food allergy [17], and were further confirmed with allergen-specific IgE testing to the most common sixteen allergens in the region [18-23]. We identified 266 patients, aged 18 years or older, with previously diagnosed atopic diseases and all cases were confirmed by at least one positive allergen-specific IgE. We also recruited healthy adults who visited the same hospital for a health check-up during the same period as potential controls. All cases and controls had a complete set of allergen-specific IgE testing, an interview with a structured questionnaire and anthropometric measurements. Final eligible controls were those who were negative in all IgE tests. Professional athletes and pregnant or breastfeeding women were excluded. Each case was matched to two healthy controls according to the following criteria: 1) from the same region (urban or rural), 2) with the same gender, and 3) within 2 years in age. We matched 532 controls to 266 atopic disease cases. All subjects provided written informed consent to participate in this study. This project was approved by the First Affiliated Hospital of Harbin Medical University’s Ethical Review Committee.

### Measurements

#### Allergen-specific IgE testing

Before matching, allergen-specific IgE in serum concentrations to sixteen most common allergens in the region were measured using the AllergyScreen system (Mediwise Analytic GmbH, Germany) in all potential cases and controls. Atopic sensitisation was defined if the concentration of at least one of the allergen specific IgE was 0.35 kU/L or greater. The sixteen allergens were *Dermatophagoides pteronyssinus*, common ragweed and mugwort, hop, cat and dog fur, egg white/egg yolk, fish, crab, shrimp, milk, beef, mutton, wheat, mould mixture (*Penicillium notatum, Cladosporium herbarum, Aspergillus fumigatus, Alternaria alternate*), tree pollen mixture (*Robur, Elm, London Plane, Willow, cottonwood*), blue mussel and German cockroach [18-23].

#### Anthropometric measurements and obese

Body weight was measured to the nearest 0.1 kg using a calibrated standard scale with participants wearing light dress without shoes. Height was measured to the nearest 0.1 cm using a stadiometer. The physician who performed the anthropometric measurements did not know the nature of this study and the grouping of the study participants. Body mass index (BMI) was calculated as body weight (kg) divided by height (m) squared (kg/m^2^). According to the Working Group on Obesity in China, the participants with a BMI≥ 28.0 kg/m^2^ were considered as obese [24].

#### Demographic, family history and lifestyle factors

A structured questionnaire interview was conducted to collect data on the characteristics of study participants. The demographic factors and other characteristics included sex, age, residential region (urban vs rural), education level and marital status. Lifestyle variables included cigarette smoking, alcohol drinking and physical exercise. The data on family history of related diseases such as eczema, asthma, hay fever and food allergy were also collected.

### Data analysis

Differences in BMI between cases and matched controls were calculated using regression for clustered data to take the matched design into consideration. The prevalence of obesity was calculated for cases and controls. To assess the associations of obesity with atopic diseases and to control for potential confounding factors, adjusted odds ratios (OR) and their 95% confidence intervals were calculated using conditional logistic regressions. A separate analysis was conducted for each of the four atopic diseases. All analyses were performed using Stata 12.1 [25].

## Results

### Characteristics of cases and controls

Table 1 shows the characteristics of total atopic cases and controls. Due to the nature of matching, two groups had identical gender and similar age distributions. The case and controls within each matched set were from the same residential region. There were no significant differences between cases and controls in height, cigarette smoking, alcohol drinking, physical exercise, education, and marital status. The proportion of participants who had a family history of atopic asthma, eczema, rhinitis, and food allergy was higher in cases than in controls. The atopic disease group also had a significantly higher prevalence of obesity than controls.

**Table 1:** Characteristics of Chinese atopic cases and their individually matched controls

	**Atopic cases**	**Controls**	**p**
Numbers	266	532	
Age, years	38.5 (12.1)	38.5 (12.0)	0.98
Males, n (%)	80 (30.1)	160 (30.1)	1.0
Residential region: rural, n (%)	37 (13.9)	74 (13.9)	1.0
Marital status: married, n (%)	213 (80.1)	441 (82.9)	0.33
Height, cm	165.4 (7.5)	165.1 (7.5)	0.54
Weight, kg	63.4 (13.2)	61.9 (10.2)	0.075
BMI, kg/m^2^	23.1 (3.9)	22.7 (3.1)	0.10
Cigarette smoking, n (%)	52 (19.5)	113 (21.2)	0.58
Alcohol drinking, n (%)	26 (9.8)	76 (14.3)	0.072
Exercise > 1 per week, n (%)	79 (31.4)	167 (31.4)	0.63
Education			0.14
Primary school	19 (7.1)	53 (10.0)	
High school	140 (52.6)	299 (56.2)	
Tertiary	107 (40.2)	180 (33.8)	
Family history of allergic diseases	96 (36.1)	119 (22.4)	<0.001
Obese, n (%)	31 (11.7)	22 (4.1)	<0.001

### BMI categories and atopic diseases

Distributions of BMI categories among cases and controls are shown in Table 2. The difference in BMI categories between atopic cases and controls reached statistical significance for the presence of any atopic disorders (p <0.001), atopic dermatitis (p = 0.020) and atopic rhinitis (p = 0.022), with cases having higher prevalence of obesity than controls. The difference between cases and controls was not statistically significant for atopic asthma and food allergy.

**Table 2:** BMI category and atopic disorders in Chinese adults

**Atopic disorders**	**BMI categories**	**Controls, n (%)**	**Cases, n (%)**	**P**
Atopic dermatitis				
	<24	122 (67.8)	59 (65.6)	0.020
	24-27.9	46 (25.6)	17 (18.9)	
	28+	12 (6.7)	14 (15.6)	
Atopic rhinitis				0.022
	<24	125 (65.8)	72 (75.8)	
	24-27.9	58 (30.5)	13 (13.7)	
	28+	7 (3.7)	10 (10.5)	
Atopic asthma				0.16
	<24	34 (63.0)	17 (63.0)	
	24-27.9	17 (31.5)	6 (22.2)	
	28+	3 (5.6)	4 (14.8)	
Food allergy				0.85
	<24	110 (67.1)	57 (69.5)	
	24-27.9	45 (27.4)	20 (24.4)	
	28+	9 (5.5)	5 (6.1)	
Any atopic disorders above				<0.001
	<24	362 (68.0)	186 (69.9)	
	24-27.9	148 (27.8)	49 (18.4)	
	28+	22 (4.1)	31 (11.7)	

### Associations of obesity with atopic diseases

Obesity was significantly associated with the presence of atopic diseases in the study population (OR = 3.2, 95% CI: 1.8, 5.7). As shown in Table 3, the associations varied for different atopic diseases. Obesity was positively and significantly associated with atopic dermatitis (OR = 2.7, 95% CI: 1.2, 6.3) and atopic rhinitis (OR = 3.1, 95% CI: 1.1, 8.7). The association between obesity and atopic asthma was positive but not significant (OR = 3.4, 95% CI: 0.6, 19.9) while the association between obesity and food allergy was weak and not statistically significant (OR = 1.1, 95% CI: 0.4, 3.7). After adjusting for potential confounding factors of age, sex, education, cigarette smoking, alcohol drinking, marital status, family history of atopic diseases and physical exercise, obesity remained significantly associated with atopic dermatitis and rhinitis.

**Table 3:** Associations of obesity with atopic diseases in Chinese adults

	**Crude**		**Adjusted **^**a**^	
**Atopic disorders**	**OR**	**P**	**OR**	**p**
Dermatitis	2.7 (1.2, 6.3)	0.023	3.5 (1.4, 8.7)	0.0078
Rhinitis	3.1 (1.1, 8.7)	0.029	4.0 (1.3, 12.3)	0.017
Asthma	3.4 (0.6, 19.9)	0.17	7.1 (0.8, 62.4)	0.078
Food allergy	1.1 (0.4, 3.7)	0.84	1.6 (0.4, 6.0)	0.47
All disorders	3.2 (1.8, 5.7)	<0.001	3.8 (2.0, 6.9)	<0.001

^a ^Adjusted for age, sex, education, cigarette smoking, alcohol drinking, marital status, family history and physical exercise.

To assess if the association between obesity and the presence of any atopic disorders depended on sex, we estimated crude and adjusted OR values of the presence of atopic disorders for obesity for males and females separately. The crude ORs were similar for males (3.1, 95% CI: 1.4, 7.2) and females (3.2, 95% CI: 1.4, 7.3), and the adjusted ORs were 4.0 (95% CI: 1.6, 9.8) for males and 3.2 (1.3, 7.8) for females.

## Discussion

In this matched case–control study, we found that obesity was significantly associated with atopic diseases in Chinese adults. Specifically, obesity was significantly associated with atopic dermatitis and atopic rhinitis, but the association between obesity and atopic food allergy was weak and not statistically significant.

In the current debate on the association between obesity and atopy in adults, some studies support the presence of a positive association [4-6]. In a study of 1997 Canadian adults, Chen et al. found a significant association between obesity and atopy with an adjusted odds ratio 1.33 (1.04, 1.71). In another study of 2090 American adults, Silverberg et al. reported positive associations of obesity with atopic dermatitis and atopic asthma [5]. On the other hand, several studies have failed to support the association between obesity and atopy in adults [7,8]. The data from Germany suggest no association between obesity and atopy (OR = 1.03, 95% CI: 0.70, 1.50) [7]. The data from Australian adults showed no association between BMI and atopy [26]. A multicentre cross-sectional survey of young adults in Europe showed that there was a positive association between high BMI and the risk of asthma attacks in women but there was no association between BMI and sensitization to any of allergens tested in the study [8]. Leung et al. showed that obesity was not associated with atopy in Chinese children [27]. Little data are available from Chinese adults. In our study, we examined the associations of obesity with four atopic diseases and our findings confirmed significant associations of obesity with atopic dermatitis and atopic rhinitis in Chinese adults.

The association between obesity and asthma was inconclusive. The sample size for assessing the association between obesity and atopic asthma in our study was too small to provide a conclusive association, as indicated by the wide 95% confidence intervals even though the point estimate of OR was as high as those for atopic dermatitis and atopic rhinitis. Our data do not support an association between obesity and food allergy.

Although our findings support a positive association between obesity and atopic diseases such as atopic dermatitis and atopic rhinitis, we are unable to establish a cause-effect relationship. Due to the nature of case–control design, all cases in this study had previously diagnosed symptomatic atopic diseases which were confirmed by positive allergen-specific IgE tests during the study period. It is possible that the observed association was due to the fact that atopic diseases increased the risk of obesity. Those with atopic dermatitis or atopic rhinitis might be more likely to experience weight gain because of restricted physical activities, increased energy intake and side effects of some treatments. Nevertheless, the positive and significant association between obesity and atopic diseases has important public health implications for control and management of both atopic diseases and obesity related chronic diseases. Our findings warrant further investigation on the causal relationship between obesity and atopic diseases and the effect of weight reduction on preventing atopic dermatitis and rhinitis in adults. As there is an increasing trend in both obesity and atopic disease in China [1,9], further understanding the underlying mechanism is important for planning intervention strategies for both obesity and atopic disorders.

There are several strengths in this study. First, all cases and controls were confirmed by allergen-specific tests to sixteen common allergens in the region to minimise potential misclassification. Second, we used an individually matched design according to multiple factors of age, sex and residential regions to improve the comparability between cases and controls. Third, data on weight and height of both cases and controls were obtained through direct physical measurements which should be more accurate and reliable than self-reported values.

There are some limitations in this study. First, because BMI was used to define obesity, we were not able to distinguish central obesity from peripheral obesity in this study. Nevertheless, several studies have consistently documented strong correlations of BMI with waist circumference and the body fat measured by dual-energy X-ray absorptiometry [28,29]. Second, the sample size was relatively small for some atopic disorders. For example, we had only 27 cases and 54 controls for assessing the association between obesity and atopic asthma. It is possible that obesity is truly associated with atopic asthma but our data had a low statistical power to detect such an association. Therefore, further studies are needed to investigate the association between obesity and asthma. Also, even though we were able to establish a positive association for some atopic disorders, due to the small sample sizes, the effect estimates were imprecise as reflected by the wide 95% confidence intervals. Further research with a large sample size is needed to more accurately quantify the association between obesity and atopic disorders in Chinese adults. Third, although we confirmed the associations of obesity with two atopic diseases (atopic dermatitis and rhinitis), we were not be able to establish the time sequence of which conditions had occurred first. Nevertheless, our findings of the associations of obesity with atopic diseases warrant further cohort studies in this area.

## Conclusions

Obesity is positively and significantly associated with atopic dermatitis and atopic rhinitis in Chinese adults, suggesting those atopic diseases are either consequences or risk factors of obesity. Our findings have important public health implications for the management and prevention of atopic diseases and obesity related chronic diseases. Further research aiming at elucidating the temporal relationship between obesity and those atopic diseases is required.

## Competing interest

The authors declare that they have no competing interests.

## Authors’ contributions

XL, ML and ZW conceived the study idea and participated in its design. XL, JX, XD, FC, ML and JS participated in the study design and conduct of data collection. XL, ML and ZW carried out statistical analysis. All authors contributed to the writing of the manuscript and critically reviewed the final version submitted for publication. All authors read and approved the final manuscript.

## Pre-publication history

The pre-publication history for this paper can be accessed here:

http://www.biomedcentral.com/1471-2458/13/12/prepub
